# Nicotine attenuates global genomic DNA methylation by influencing DNMTs gene expression in human endometrial stromal cells

**DOI:** 10.1186/s41021-020-0144-5

**Published:** 2020-02-05

**Authors:** Fatemeh Zal, Amir Yarahmadi, Hamidreza Totonchi, Mahdi Barazesh, Mostafa Moradi Sarabi

**Affiliations:** 1 Department of Biochemistry, School of Medicine, Shiraz University of Medical Sciences, Shiraz, Iran; 2 Infertility Research Center, Shiraz University of Medical Sciences, Shiraz, Iran; 3 Biotechnology Department, School of advanced medical sciences and technologies, Shiraz University of Medical Sciences, Shiraz, Iran; 4 Department of Biochemistry and Genetics, School of Medicine, Lorestan University of Medical Sciences, Khorramabad, 381251698 Iran; 5 Razi Herbal Medicines Research Center, Lorestan University of Medical Sciences, Khorramabad, Iran; 6 Hepatitis Research Center, Lorestan University of Medical Sciences, Khorramabad, Iran

**Keywords:** Nicotine, DNMTs gene expression, Global DNA methylation, Endometrial cancer

## Abstract

**Background:**

There is increasing evidence indicating an incidence of infertility and also the risk of endometrial cancers among smokers. However, the mechanism underlying nicotine adverse effect on female reproduction remains unclear. Growing evidence has suggested that environmental exposures such as nicotine could modulate the epigenome. No study has yet been published to evaluate the direct effect of nicotine on the epigenome profiling of human endometrial stromal cells (HESC). Herein, we decided to examine the direct effects of nicotine on global genomic DNA methylation status and DNA methyl- transferases (DNMTs) gene expression in HESC. HESC were treated with different doses of nicotine (0 or control, 10^− 11^, 10^− 8^ and 10^− 6^) M for 24 h and their genomic global DNA methylation and gene expression of DNMTs (DNMT1, DNMT3A, and DNMT3B) were investigated using ELISA and real-time PCR, respectively.

**Results:**

Nicotine treatments reduced the average level of DNMTs gene expression by 90, 79, and 73.4% in 10^− 11^, 10^− 8^ and 10^− 6^ M of nicotine treated cells as compared to control cells, respectively (*p* < 0.05). Also, 10^− 8^ and 10^− 6^ M of nicotine concentrations effectively reduced the amounts of 5-methylated cytosine (5-mC) by 1.09 and 1.87% compared to control cells, respectively (*p* < 0.05). The 5-mC percentages were positively correlated with the relative cellular DNMTs expression in HESC as verified by the Pearson correlation test.

**Conclusion:**

An interesting possibility raised by the current study is that the reduced genomic global DNA methylation level in HESC may be partly due to the suppression of DNMTs gene expression caused by nicotine in these cells.

**Graphical abstract:**

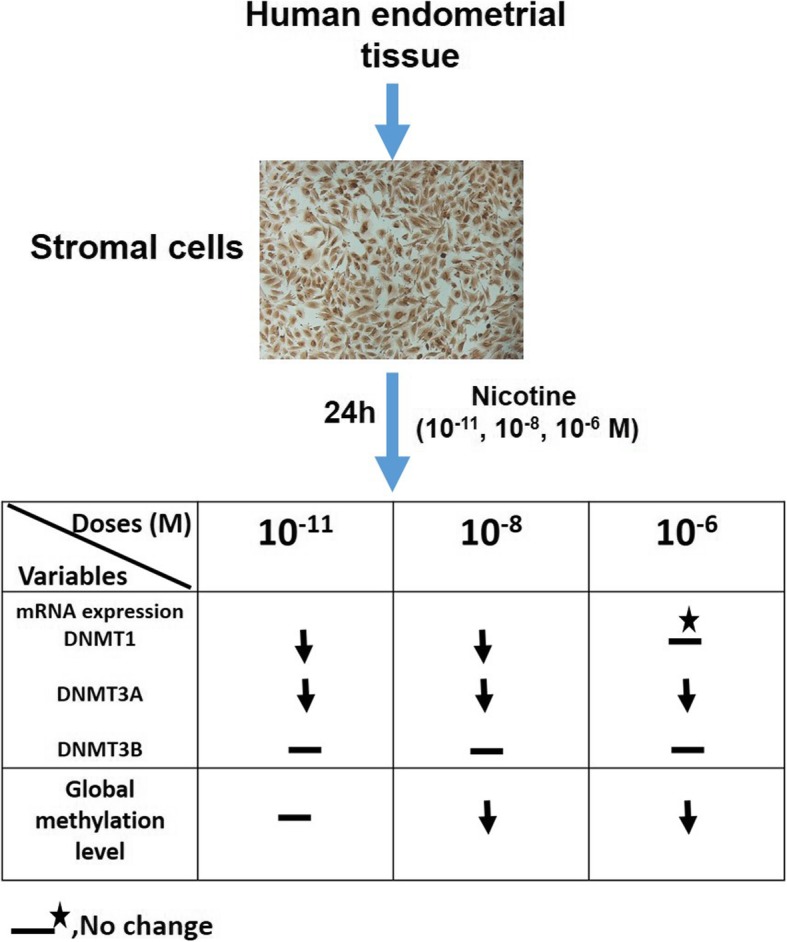

## Introduction

DNA methylation is described as an epigenetic mechanism involving the transfer of methyl group at the 5′ carbon of cytosine nucleotides to form 5-methyl cytosine (5-mC) in CpG islands and modulates gene expression [1–3]. It has been suggested that three active forms of DNA methyltransferases (DNMT) including DNMT1, DNMT3A and DNMT3B are responsible for maintenance and generation of DNA methylation [4]. DNMT1 known as maintenance methyltransferase, is involved in lifelong maintenance of DNA methylation during processes such as cell division and ubiquitously expressed in proliferative cells [5, 6]. DNMT3A and DNMT3B known as de novo methyltransferases and have DNA methyltransferase activity without any template and they can introduce methylation into naked DNA [7, 8]. Alteration in DNA methylation and elevated expression level of DNMTs are suggested the potential mechanisms in malignancies [9]. Previous studies showed that global DNA hypomethylation takes place in many human cancers [10, 11]. Furthermore, the overall loss of genomic DNA methylation has been proposed to be an important screening marker for carcinogenesis [12, 13]. Nowadays, environmental exposures such as tobacco smoking is recognized as a health hazard and the main cause of many human diseases including different types of cancer [14–16]. Cigarette tobacco comprises about 4000 compounds which many of them belonging to chemical substances such as alkaloids [17–19]. Nicotine, is the principle tobacco alkaloid and represents more than 95% of total alkaloid in cigarette smoke [20]. Moreover, nicotine poses several health hazards including cell proliferation, DNA mutation, ill impacts on the reproductive health and other different cellular pathways which lead to cancer [21–23]. It is speculated by some researchers that the expression levels of DNMTs are remarkably increased in various cancers and DNA methylation can occur following exposure to exogenous stimuli such as smoking [8, 24]. Moreover, other approaches highlight that cigarette smoking in the context of both current smoking and prenatal exposure may influence DNMTs activity and is a strong modifier of DNA methylation [8, 25]. Various epidemiological studies demonstrated a predictable and significant incidence of infertility and an increased risk of spontaneous abortion among smokers [26, 27]. However, the mechanism underlying tobacco’s adverse effect on female reproduction remains unclear [15, 28]. No study has yet been published to evaluate the direct effect of nicotine on the DNA methylation profiling of human endometrial stromal cells (HESC). Based on these data and owing to the increasing use of cigarette smoking among women, we have investigated the effect of nicotine on HESC for assessing the carcinogenic effects of nicotine on the epigenetic profiling including DNMTs transcription levels and global DNA methylation in these cells.

## Material and methods

### Materials

Nicotine was purchased from Sigma Chemical Co (Poole, Dorset, UK); DMEM, FCS (fetal calf serum), penicillin, streptomycin were obtained from Gibco-BRL (Paisley, UK). Trypsin was from BDH-England. 5-mC DNA enzyme-linked immunosorbent assay (ELISA) kit was purchased from Zymo Research (Freiburg, Germany). Tripure RNA isolation reagent was from Roche Applied Sciences (Indianapolis, Indiana, USA). cDNA (complementary DNA) synthesis kit was purchased from Fermentas Life Science (Waltham, Massachusetts, USA). A real-time PCR master mix was obtained from amplicon (Odense, Denmark).

### Sample preparation

Human endometrial biopsies were acquired from women within reproductive age fertile (aged between 20 and 40 years) which had normal menstrual cycles and undergoing sterilization procedures. All experimental and surgical procedures were approved by the committee of investigations involving human participants of Shiraz University of Medical Sciences. Informed consent was acquired from a subject before the collection of any tissue samples for this study. None of the subjects had used any hormonal contraception within the 3 months before the operation. A part of the removed endometrium was fixed for histological examination to confirm that the taken tissue was normal.

### Immunocytochemistry staining

The identification of HESC were performed by the method as previously described [29, 30]. Five healthy biopsies were collected and all tissues cut into 2- to 3-mm pieces, then all 5 biopsies were pooled to make a single cell suspension which was then expanded to get enough cells for the experiment. Biopsies incubated with 1 mg/mL of collagenase type 1 in DMEM/ 10% FCS with stirring at 37 °C for 2 h. The suspension was then filtered through a 40-mm nylon sieve that only allowed the stromal cells to pass through, but the intact glands were retained. At the end of the isolation procedure, the cells were counted with a hemocytometer and cellular viability was determined by the trypan blue staining. Representative cells were then stained with vimentin, a marker to ensure that the monolayers comprised of 98% or more of stromal cells.

### Cell culture procedure and treatments

The endometrial cells extracted from biopsies were cultured in Dulbecco Modified Eagle Medium (DMEM) supplemented with 10% FCS, 100 IU/mL penicillin, and 100 mg/mL streptomycin and then incubated in a humidified atmosphere of 5% CO_2_ and 95% air at 37 °C. The medium was changed every 3 to 4 days. All experiments were done using cells in the passage numbers 2 to 5 at more than 70% confluency. Cells were treated by adding 0 (control), 10^− 11^, 10^− 8^ and 10^− 6^ M of nicotine, respectively. All treatments were performed in triplicate within each experiment. Medium pH was not affected by the addition of any concentration of nicotine.

### RNA extraction and real-time PCR

Total RNA was extracted from HESC using the TriPure isolation reagent (Roche Applied Science, Indianapolis, Indiana, USA). Before cDNA synthesis, the integrity of purified RNA was assessed by electrophoresis on 1.5% agarose gel visualized by Gel-Red staining. Then 2 μg of purified total RNA was used for cDNA synthesis using revert aid first-strand cDNA synthesis kit (Fermentas Life Science, USA) following the manufacturer’s instruction. Expression levels of DNMT1, DNMT3A, DNMT3B, and β-actin as a reference gene were determined by quantitative Real-Time PCR assay using SYBR Green master mix (Amplicon, Denmark) with an ABI 7500 detector (Applied Biosystems, USA). The primers information used for real-time PCR is described in Table 1. After an initial pre-cycling heat activation for 10 min at 95 °C, the samples were amplified for 40 cycles; denaturation at 95 °C for 15 s, annealing at 60 °C for 1 min, extension at 72 °C for 1 min. The duration of the final extension reaction was increased to 10 min at 72 °C to allow the completion of reaction products. The genes’ relative expression levels were determined using the 2^−ΔΔCT^ standard methods [31].

**Table 1:** Primers’ sequence used for quantitative RT-PCR

Gene	Forward primer	Reverse primer	Product size (bp)
DNMT1	5′-TACCTGGACGACCCTGACCTC-3′	5′-CGTTGGCATCAAAGATGGACA-3′	103
DNMT3A	5′-TATTGATGAGCGCACAAGAGAGC-3′	5′-GGTGTTCCAGGGTAACATTGAG-3′	111
DNMT3B	5′-GGCAAGTTCTCCGAGGTCTCTG-3′	5′-TGGTACATGGCTTTTCGATAGGA-3′	113
β-Actin	5′-AATCGTGCGTGACATTAAG-3′	5′-GAAGGAAGGCTGGAAGAG-3′	101

### Genomic DNA preparation

Genomic DNA was extracted from the HESC by the standard method of proteinase K digestion, phenol-chloroform extraction and ethanol precipitation described previously [32].

### Analysis of global DNA methylation

Global genomic DNA methylation in DNA isolated from HESC was assayed by measurement of 5-methylated cytosines (5-mC) using the ELISA method as per the manufacturer’s protocols (Zymo Research, Germany). The amount of 5-mC was proportional to the optical density (OD) intensity measured at 450 nm. All of the measurements were performed in triplicate within each experiment. For more information please refer to our previously published article [2].

### Statistical analysis

We used SPSS 18 analytic software (SPSS, Inc., Chicago) and GraphPad Prism (Version 8·01) for data analysis. Data, presented as mean ± SD, were analyzed using one-way ANOVA followed by Tukey’s multiple comparison tests. Differences with *p* < 0.05 were set as the level of significance.

## Result

**Fig. 1:**
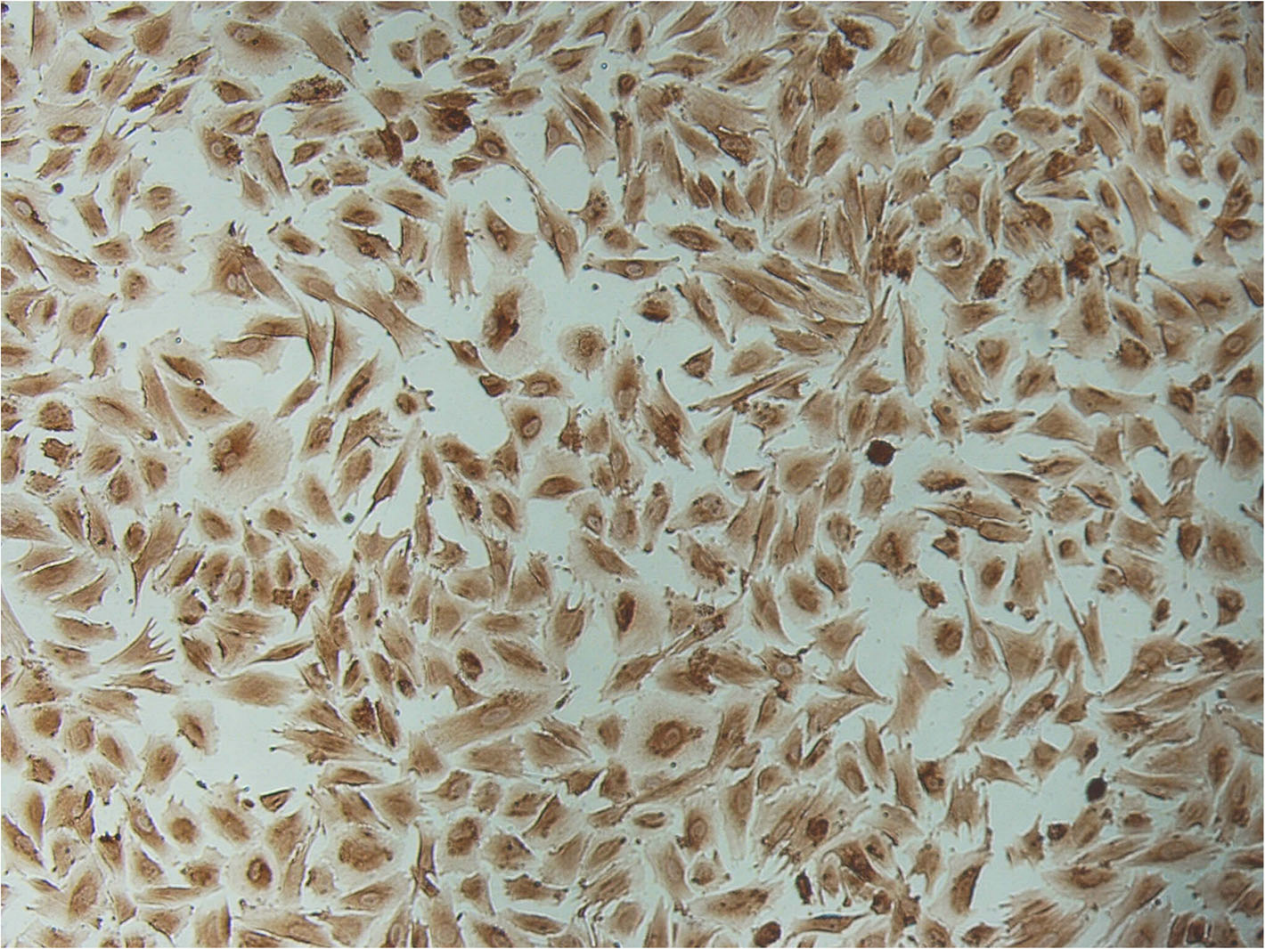
Phase-contrast pictures of Immunocytochemistry assay: strong staining was seen with primary antibody to vimentin as a marker for stromal cells (200× magnification)

In the present study, we used three different concentrations (10^− 11^, 10^− 8^ and 10^− 6^ M) of nicotine to determine the effect of nicotine treatments on global DNA methylation and DNMT1,-3A and -3B gene expression in HESC separated from women undergoing sterilization procedures. At first, with vimentin staining, we confirmed that the monolayers comprised 98% or more stromal cells (Fig. 1).

### Effect of nicotine treatments on DNMTs gene expression

For assessment of the influence of nicotine treatments on the DNMT1, DNMT3A, and DNMT3B gene expression, HESC were treated with different doses of nicotine as described in the “Material and Methods” section. The gene expression level of DNMT1, DNMT3A and DNMT3B in the nicotine treated cells were quantified and compared with those in the control group. Finally, the average expression levels of DNMTs were calculated from the combined expression values for each group and represented as mean ± SD. To confirm that there was no contaminating of genomic DNA during the amplification process, minus RT control PCR reactions were conducted. There was no amplification product detected for any specific pair of primers used in these reactions.

**Fig. 2:**
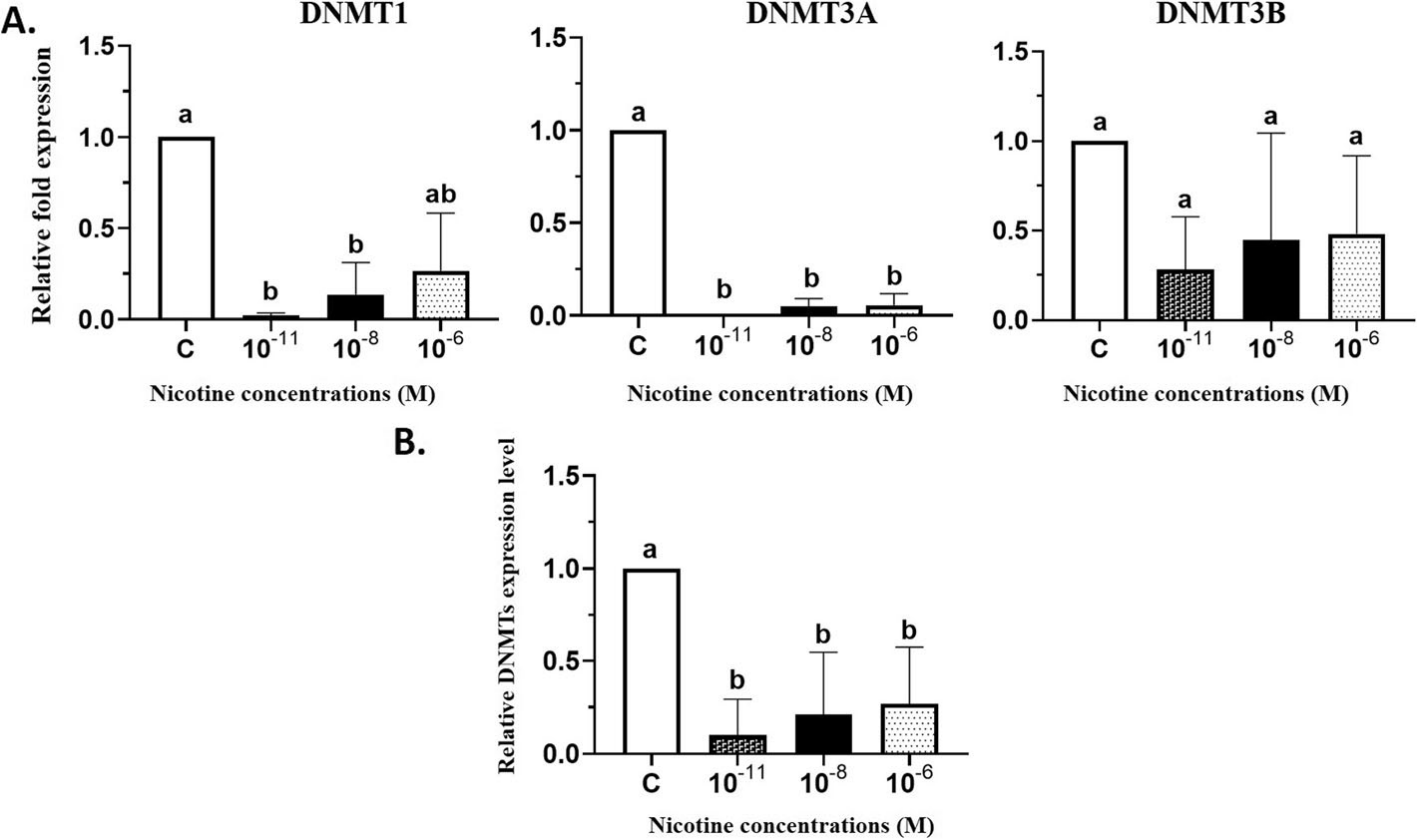
**A.** Comparison of the relative expression of DNMT1, DNMT3A and DNMT3B in human endometrial stromal cells that were treated with 10^− 11^, 10^− 8^ and 10^− 6^ M of nicotine, measured by quantitative Real-time-PCR. Expression of each gene was normalized to β-Actin mRNA. The culture-media treated control cells were used as a reference, whose expression levels were set to 1.0, and expressions in all other cells were expressed as an n-fold difference relative to controls. Mean values ± SD of three experiments are given. Bars marked with different letters are significantly different as verified by Tukey’s honestly significant difference multiple comparison test (*p* < 0.05). **B.** A summary of the change in the average expression of all three DNMTs (T1/3A/3B) in HESC treated with 10^− 11^, 10^− 8^ and 10^− 6^ M of nicotine (*p* < 0.05)

As shown in Fig. 2a, all of the three nicotine concentrations (10^− 11^, 10^− 8^ and 10^− 6^) M caused a reduction by 98, 86.5 and 73.5% in DNMT1 gene expression respectively, but it was significant only at 10^− 11^ and 10^− 8^ M doses of nicotine (*p* < 0.05). Furthermore, more than 90% reduction in DNMT3A gene expression was observed in HESC treated with 10^− 11^, 10^− 8^ and 10^− 6^ M of nicotine (*p* < 0.05) (Fig. 2a). In all three doses, nicotine demonstrated higher efficacy on reduction (> 90%) of DNMT3A in HESC. Notably, there was no difference in mRNA transcription level for DNMT3B in HESC after all three doses of nicotine treatments compared with control cells (Fig. 2a). However, our results indicated that the average expression levels of all three DNMTs (T1/3A/3B) effectively reduced by 90, 79, and 73.4% in 10^− 11^, 10^− 8^ and 10^− 6^ M doses of nicotine treated endometrial stromal cells, respectively (*p* < 0.05) (Fig. 2b).

### Effect of nicotine treatments on global genomic DNA methylation

**Fig. 3:**
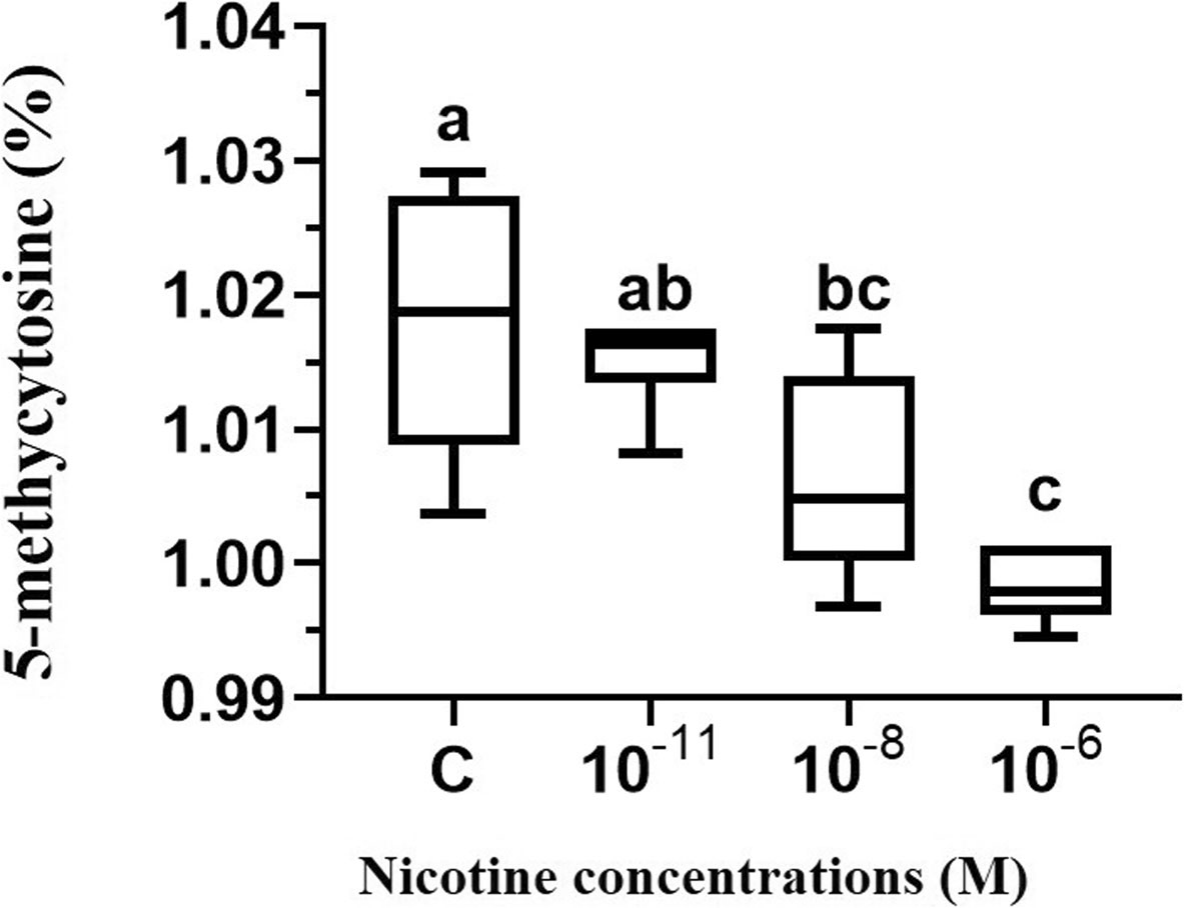
Effects of nicotine at the concentrations of 10^− 11^, 10^− 8^ and 10^− 6^ M on global DNA methylation in HESC. Values represent mean ± SD of three experiments. The Bars marked with different letters represents significantly different from other samples as verified by Tukey’s honestly significant difference multiple comparison test (*p* < 0.05)

**Fig. 4:**
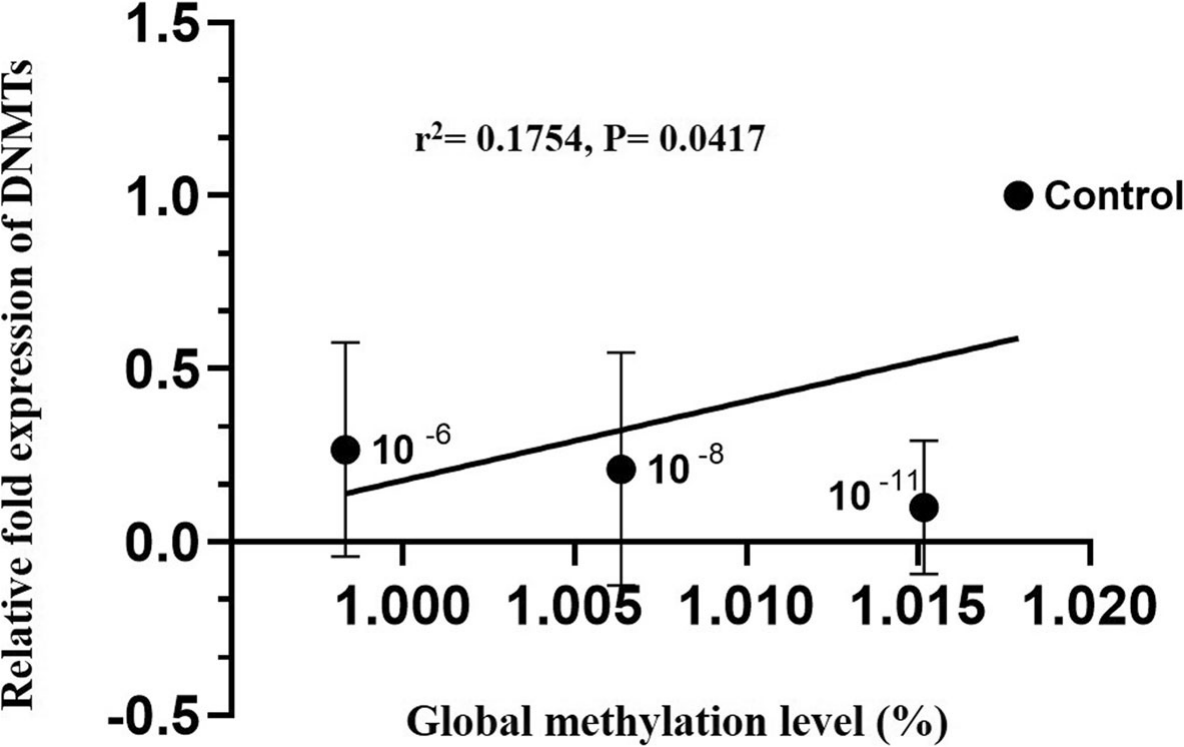
Correlation of average expression levels of DNMT1, DNMT3A and DNMT3B with global DNA methylation in 10^− 11^, 10^− 8^ and 10^− 6^ M concentrations of nicotine treated human endometrial stromal cells. Correlation between mean expressions of DNMT1/3A/3B and %5-mC in genomic DNA of HESC was verified by Pearson correlation test (r^2^ = 0.1754, *p* = 0.0417)

To evaluate the effect of nicotine treatments on the amount of DNA methylation, we also used three doses including 10^− 11^, 10^− 8^ and 10^− 6^ M of nicotine on genomic global DNA methylation in HESC. As shown in Fig. 3, Our results demonstrated that overall about 1.015, 1.006, 0.998, and 1.017% of the cytosines were methylated in the genomic DNA of HESC exposed to 10^− 11^, 10^− 8^ and 10^− 6^ M of nicotine, and control group, respectively. But the differences in the amount of global DNA methylation were significant only at 10^− 8^ and 10^− 6^ M doses of nicotine in comparison to the control group (*p* < 0.05) (Fig. 3). Interestingly, the Pearson correlation analyses indicated significant positive correlation between mean DNMT1/3A/3B gene expression level and percentage of 5-mC in HESC (Pearson’s correlation; r^2^ = 0.1754, *p* = 0.0417) (Fig. 4). Overall, our results indicated that the treatment of nicotine coordinately reduced the expression of DNMTs as well as global DNA methylation in HESC.

## Discussion

Characterizing the molecular mechanisms by which nicotine affects gene regulation will be beneficial to understand how its adverse effects happen in the human reproductive system. Epigenetic mechanisms such as aberrant DNA methylation is the most common epigenetic change associated with human disease such as cancer [8]. Also, DNA methylation controls many important processes including embryonic development, proliferation, differentiation, and gene expression [33, 34]. Clinical and experimental studies have demonstrated that environmental exposures, such as cigarette smoking, which contains carcinogenic alkaloids like nicotine can alter DNA methylation in any stage of human development. Although various in vitro and in vivo studies have demonstrated that nicotine could alter DNMT expression in mouse brain neurons and esophageal epithelial cells, no study has yet been published to examine the direct effect of nicotine on the DNA methylation profiling in HESC. Therefore, we investigated the direct effects of three doses (10^− 11^, 10^− 8^ and 10^− 6^ M) of nicotine on global genomic DNA methylation and DNMTs gene expression in HESC. The rationale for using 10^− 11^, 10^− 8^ and 10^− 6^ M concentrations were that we have reported previously that the nicotine doses below 10^− 6^ M are safe for using in the in-vitro model of human cells [30, 35]. Our results indicated that treatments of HESC with 10^− 11^ and 10^− 8^ M of nicotine significantly decreased the level of DNMT1 gene expression by 98 and 86.5% in comparison to control cells (Fig. 2a). Previous studies reported that nicotine induced a significant decrease of DNMT1 gene and protein expression in the mouse brain neurons and human esophageal squamous epithelial cells [36, 37]. It has been documented that nicotine can bind to the cellular nicotinic acetylcholine receptors and thus increases intracellular calcium and leads to activation of cAMP cascade and also related response element-binding protein and subsequent downregulate DNMTs [36, 38]. Moreover, we found a higher efficacy of all three doses of nicotine in the reduction of DNMT3A gene expression in HESC (> 90% reduction) (Fig. 2a). Also, we found no difference in gene expression level of DNMT3B in HESC (Fig. 2a). However, we observed a trend for coordinate patterns of three DNMTs expressions and that nicotine effectively reduced the average expression levels of DNMTs (T1/3A/3B) in HESC (*p* < 0.05) (Fig. 2b). It has been shown that the expression levels of DNMTs positively correlate with each other in women with endometriosis [39]. Other studies have been also suggested that the expression of DNMTs is disrupted in endometriosis [39]. Aberrant DNA methylation may play an essential role in the pathogenesis of endometriosis and endometrial cancer [40]. However, the role of DNMTs gene expression in endometriosis has been controversial. Many studies reported the up-regulated expression of only for the DNMT3A and not for DNMT1 and DNMT3B in the endometriotic tissue which leads to hypermethylation in endometriosis [40, 41]. Other studies reported the lower expression of DNMTs in women with endometriosis as compared to disease-free controls [42, 43]. Also, we found that treatments with 10^− 8^ and 10^− 6^ M doses of nicotine significantly reduced methylated 5-mC by 1.09 and 1.87% in HESC (*p* < 0.05) (Fig. 3). Moreover, we found significant positive correlation between mean DNMT1/3A/3B gene expression level and percentage of 5-mC in HESC (Pearson’s correlation; r^2^ = 0.1754, *p* = 0.0417) (Fig. 4). An interesting possibility raised by our study is that induced DNA hypomethylation by nicotine may be mediated by down-regulation of DNMTs. Extensive data indicated that global DNA hypomethylation is associated with a poor prognosis, tumor initiation and progression in different types of cancer including endometrial carcinoma [44–47]. It has been suggested that nicotine alter the epigenome and DNA methylation through putative different possible mechanisms including: (1) Via DNA damaging and subsequent recruitment of DNMTs(2) By activating of cAMP cascade and downregulation of gene expression (3) By modulating of activity of DNA-binding factors such as SP1, that binds to C_P_G islands and plays an important role in DNA methylation of CpG islands in gene promoters (4) Via hypoxia and activation of HIF-1α and subsequent synthesizes *S*-adenosylmethionine (SAM), a major biological methyl donor that provides methyl group for DNA methylation reactions [38, 48–51].. Further in vitro and in vivo studies are required to characterize the effects of nicotine on DNMTs gene expression in global and gene-specific DNA methylation in human reproduction and endometrial cells.

## Conclusion

In this study, we have demonstrated that nicotine can alter the DNMTs gene expression and global DNA methylation in HESC. Our study provides significant insight regarding the role of epigenetic mechanism of nicotine-mediated regulation of gene expression and DNA methylation in the pathogenesis of endometrial cancer and female reproduction in endometrial cells.

## Data Availability

All data included in this study are available upon request by contacting with the corresponding author.
